# Designing and evaluating the children’s developmental motor disorders system: an experience from a developing country

**DOI:** 10.1186/s12911-023-02223-2

**Published:** 2023-07-17

**Authors:** Elahe Gozali, Reza Safdari, Bahlol Rahimi, Marjan Ghazisaeedi, Hamidreza Farrokh-Eslamlou, Malihe Sadeghi

**Affiliations:** 1 Health and Biomedical Informatics Research Center, Urmia University of Medical Sciences, Urmia, Iran; 2 Health Information Management and Medical Informatics Department, School of Allied Medical Sciences, Tehran University of Medical Sciences, Tehran, Iran; 3 Department of Health Information Technology, School of Allied Medical Sciences, Urmia University of Medical Sciences, Urmia, Iran; 4 Department of Public Health, School of Health, Reproductive Health Research Center Clinical Research Institute, Urmia University of Medical Sciences, Urmia, Iran; 5 Department of Health Information Technology, Sorkheh School of Allied Medical Sciences, Semnan University of Medical Sciences, Semnan, Iran

**Keywords:** Motor Disorders, Child, Software Design, Evaluation, Requirements, Registries, Health Care systems

## Abstract

**Background:**

Developmental disorders are a prevalent problem in the health sector of low- and middle-income countries (LMICs), and children in these countries are at greater risk. A registry system is helpful and vital to monitoring and managing this disease.

**Objective:**

The present study aims to develop an electronic registry system for children's developmental motor disorders.

**Methods:**

The study was conducted between 2019 and 2020 in three phases. First, the requirements of the system were identified. Second, UML diagrams were first drawn using Microsoft Visio software. Then, the system was designed using the ASP.NET framework in Visual Studio 2018, and the C# programming language was used in the NET 4.5 technology platform. In the third phase, system usability was evaluated from the users' viewpoint.

**Results:**

The findings of this research included system requirements, a conceptual model, and a web-based system. The client and system server connection was established through the IP/TCP communication protocol in a university physical network. End users approved the system with an agreement rate of 87.14%.

**Conclusion:**

The study's results can be used as a model for designing and developing systems related to children's developmental movement disorders in other countries. It is also suggested as a valuable platform for research and improving the management of this disease.

**Supplementary Information:**

The online version contains supplementary material available at 10.1186/s12911-023-02223-2.

## Introduction

Childhood is the base and foundation of learning and building a successful future, provided that the child's development is done correctly [1]. Neurodevelopmental disorders in children are characterized by early disabilities or defects in language, communication, cognitive, motor, and social skills [2]. Developmental disabilities occur when children do not show the significant developmental characteristics expected based on age in the mentioned areas [3, 4]. These disabilities and disorders affect several areas of life. It affects and leads to disruption in the functioning of daily life [2], such as self-care, academic activities, and motor activities [5], and lower academic performance results in less employment and reduced income-earning skills [6].

After infections and trauma, developmental disorders are the most common problems in pediatric medicine, and half of these disorders are not detected until school age and are not treated [7, 8]. This disorder is found in five to 10% of school-age children. It is one of the most common disorders in this age group [9]. Its prevalence in the pediatric population is estimated at between 5 and 15% globally [4]. According to an estimation in 2016, about 52.9 million children under five worldwide suffered a developmental disability [10]. In the United States, developmental delay is common in children under one year of age [11], and its rate has shown a significant increase in children aged 3 to 17 years between 2009 and 2017 so that a developmental disability was reported in one out of every six children [12]. Among children aged 0 to 6 years in Taiwan from 2000 to 2015, the rate increased from 2.0% to 5.7% [13], and in the Northeast, this rate was 11.36% [14]. It increased from 6.9% to 7.42% in Australia between 2009 and 2015 [15]. In low- and middle-income countries (LMICs), this rate is higher than in other parts of the world, and children in these countries are at greater risk [6], so 33% of these children have developmental disabilities, and 43% of them are deprived of their growth potential due to poverty, socio-demographic and environmental factors [16].

In the Middle East, the rate of developmental disabilities increased between 2010 and 2016 [10]. Its rate is generally 18 to 22% in Iran, a developing country in the Middle East region [17], and 13.98% in its various cities in the northwestern parts [6]. Diagnosing disorders under five is considered a golden opportunity, and regular monitoring of the children's condition is considered a key measure for diagnosis. Monitoring requires registering data related to children's development to control and follow up [18]. Registries provide an opportunity to address challenges in estimating the prevalence, including changing labeling practices and definitions. Updated estimates can also improve diagnosis and increase access to services in the prevalence of diagnosed developmental disabilities [12]. From another viewpoint, the large volume of medical data and their accumulation is a challenge requiring accurate data management and evaluation [19]. Registry systems are one of the important tools of data management and supplement cross-sectional studies that determine the difference in disease rates with longitudinal studies [20]. These systems use the methods of observational studies to collect similar clinical data to evaluate predictions and the results of measures in defined populations of a specific disease, a condition, or people who have been exposed to an event [21]. These systems have the potential to facilitate the management of patients and their ill conditions [19] and use their data to determine the incidence rate and the course of diseases [20]. They also provide primary information as a preliminary evaluation for policy-making purposes [21].

Since the data of children with developmental disorders are collected manually in most centers in Iran, and there are many challenges regarding the information of these patients and the management of this disease due to the lack of accurate and timely information at the macro level, it is necessary to use an electronic registry system to address this challenge. Hence, the present study aims to design and evaluate the national system of registering and monitoring children's developmental motor disorders based on the web in Iran.

## Materials and methods

The present study was conducted as a project and in three phases. The first phase of this project and its results were published in the previous article [22]. An abstract of the first phase and other phases of the project is presented in the present study. The present study was a descriptive-developmental study conducted with an application from 2017 to 2019.

### First phase: Development of system requirements

The rules and guidelines published by the Ministry of Health and Medical Education and Maternal and Newborn Health Centers were examined in this phase. Then, a database search was done using the relevant keywords between 2010 and 2019, and the extracted English articles were reviewed. The obtained information was used to develop the questionnaire and select the system requirements. This questionnaire included five sections with 140 questions based on a five-point Likert scale. The reliability of the questionnaire was calculated by Cronbach's alpha coefficient of 0.89. Ten experts in the fields ( two pediatric neurology, two obstetrics and gynecology, two maternal and child health, two health information management, and two medical informatics), approved the content validity of the questionnaire. Data were analyzed using SPSS-20 software (IBM Corporation, New York, USA). The previous study presents this project phase in detail [22].

### Second phase: Designing and creating the system

In the second phase, using the results of the first phase of the study, as well as the presence of the researcher in the specialized clinic for child growth and development and observing the work process, as well as holding brainstorming sessions with the research team, the conceptual model of the system was drawn in the form of scenarios, applied diagrams, class, the sequence and activity using Microsoft Visio software and the research team finalized it. Then, the system was created according to the conceptual model and using the ASP.NET framework on the server. The system was designed in the Visual Studio-2018 software using C# programming language in the NET 4.5 technology platform for high-level programming and user interface, and SQL Server database, which is a relational database, was used to store data.

### Third phase: System evaluation

In this phase, after implementing the system as a pilot, the system was evaluated in two steps. The researcher evaluated the accuracy of the system's performance in the first step. In this step, 20 medical records of children with developmental motor disorders were randomly selected, and their information was entered into the system. In the second step, the system's usability was evaluated from the end user's viewpoint. In this step, the statistical population of the study consisted of experts and specialists in health information management and medical informatics working in the paramedical faculty of Urmia University of Medical Sciences (5 people) and specialists in children's developmental motor disorders at Urmia University of Medical Sciences in 2019 (9 people) who had evaluation experience in the field of health information systems. A questionnaire was used to evaluate the system (Supplementary file). This questionnaire was developed based on a combination of well-known software quality evaluation models, including the McCall [23], Boehm [24], and Clements Kazman models [25]. The Ministry of Health, Treatment, and Medical Education approved the validity and reliability of this questionnaire. This questionnaire included six sections and 39 questions in the two-option form (yes or no). The first section of the questions related to the data content of the electronic system of registering and monitoring children's developmental motor disorders (including 8 questions). The second section was related to questions about the physical design of the system (including 9 questions), the third section was related to questions about the technical characteristics of the electronic system of registering and monitoring children developmental motor disorders (including 7 questions), the fourth section was related to questions about the system flexibility (including 5 questions), the fifth section was related to questions about the system participation (including 5 questions), and the sixth section was related to questions about the system user support (including 5 questions). The research subjects were requested to use the system for at least two weeks and then complete the system usability questionnaire. The answers were analyzed descriptively (calculation of frequency and percentage). After collecting the data, they were entered into SPSS-22 software and analyzed using statistical methods (frequency and percentage).

## Results

According to the results, the requirements of the children's DMD registry system were identified in three areas, including demographic data elements (24 data elements), clinical data elements (87 data elements), and technical requirements (28 capabilities). Its results have been presented in the previous article [22]. Unified modeling language diagrams were used to model the system of registering and monitoring children's developmental motor disorders.

**Fig. 1:**
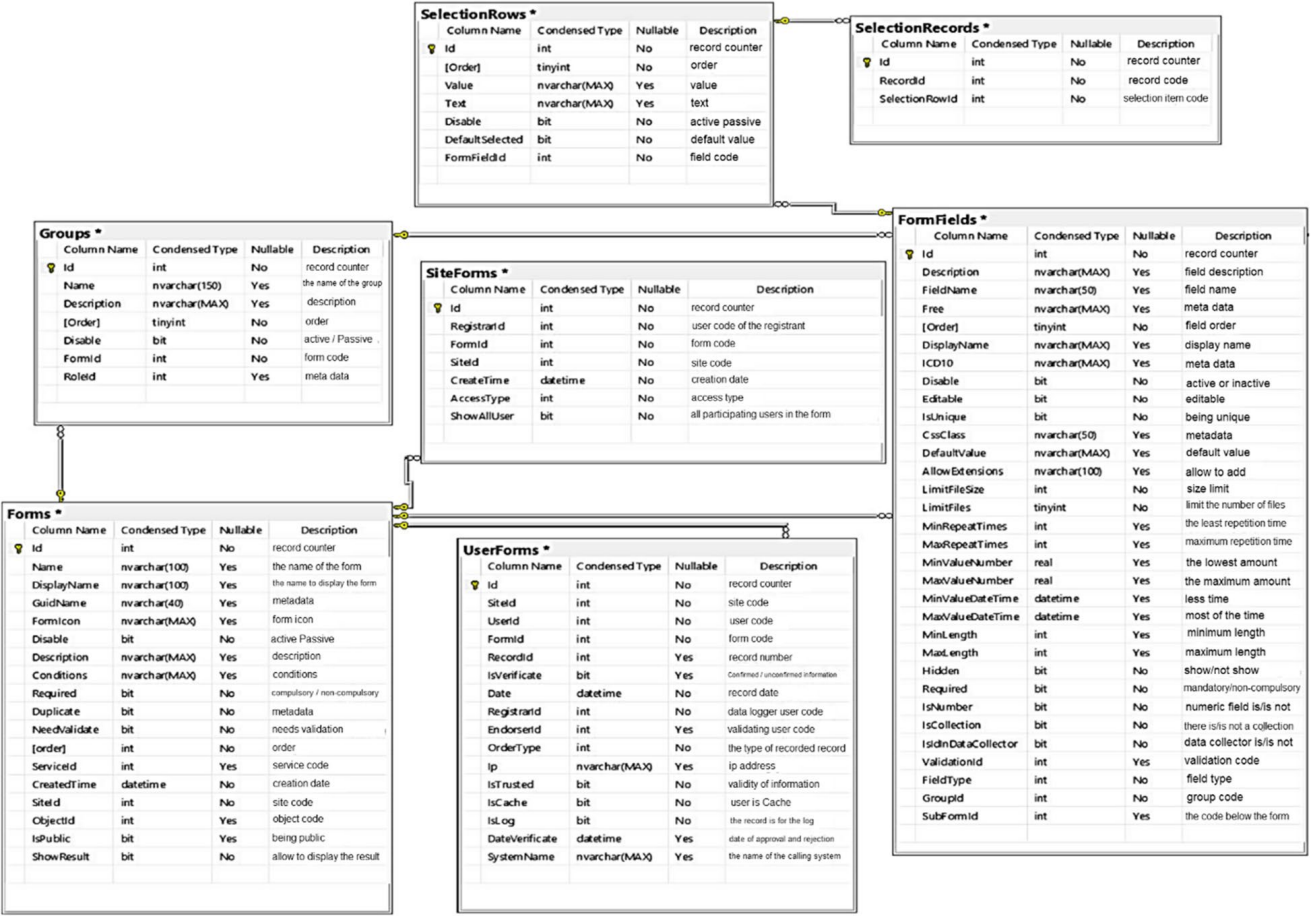
class diagram of Forms creation

**Fig. 2:**
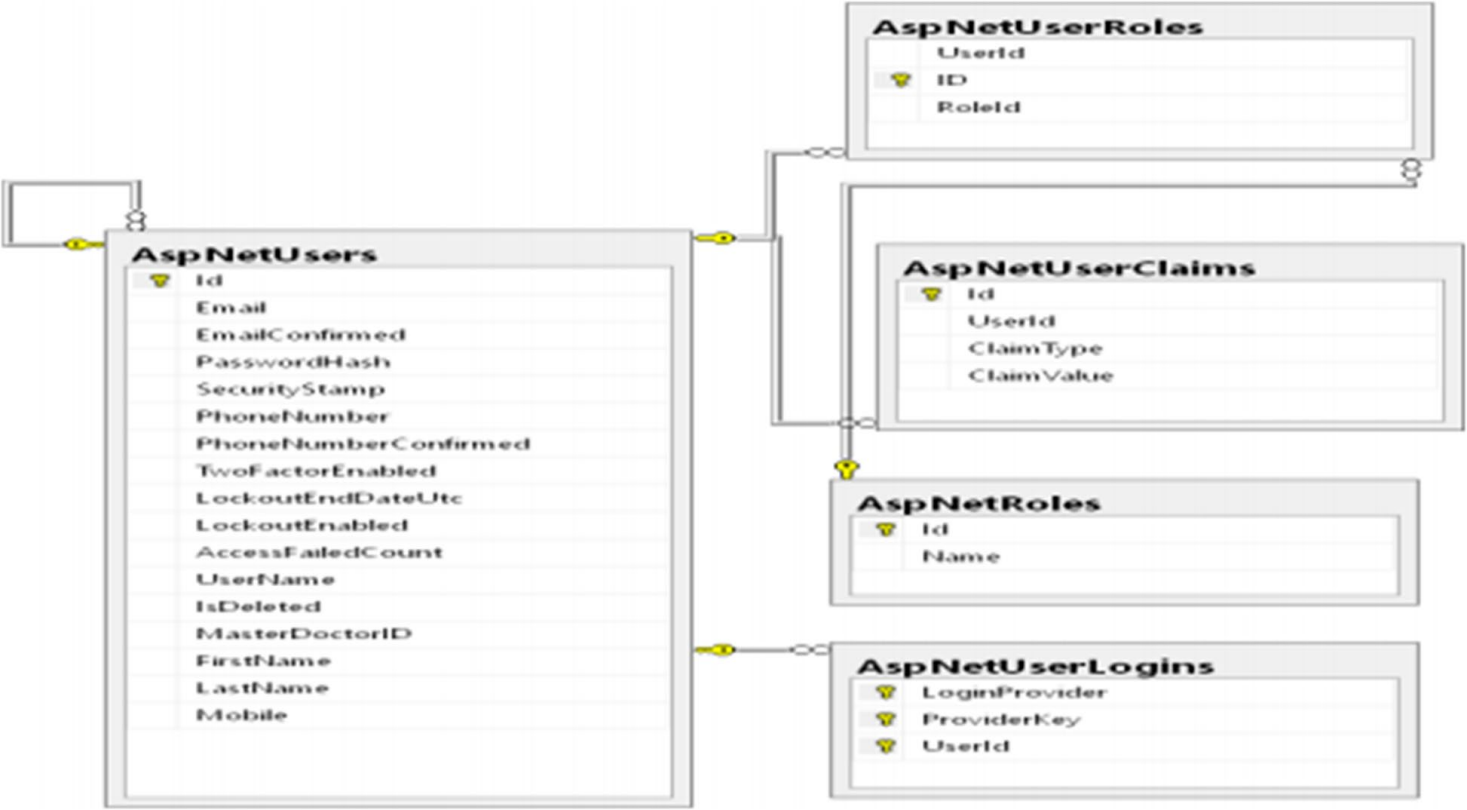
Class diagram of System user identification

Using Microsoft Visio-2016 Software, scenario tables, application diagrams, sequence, activity and system class were developed. For example, the form creation class diagram (Fig. 1) and the system user identification class diagram (Fig. 2) have been presented.

**Fig. 3:**
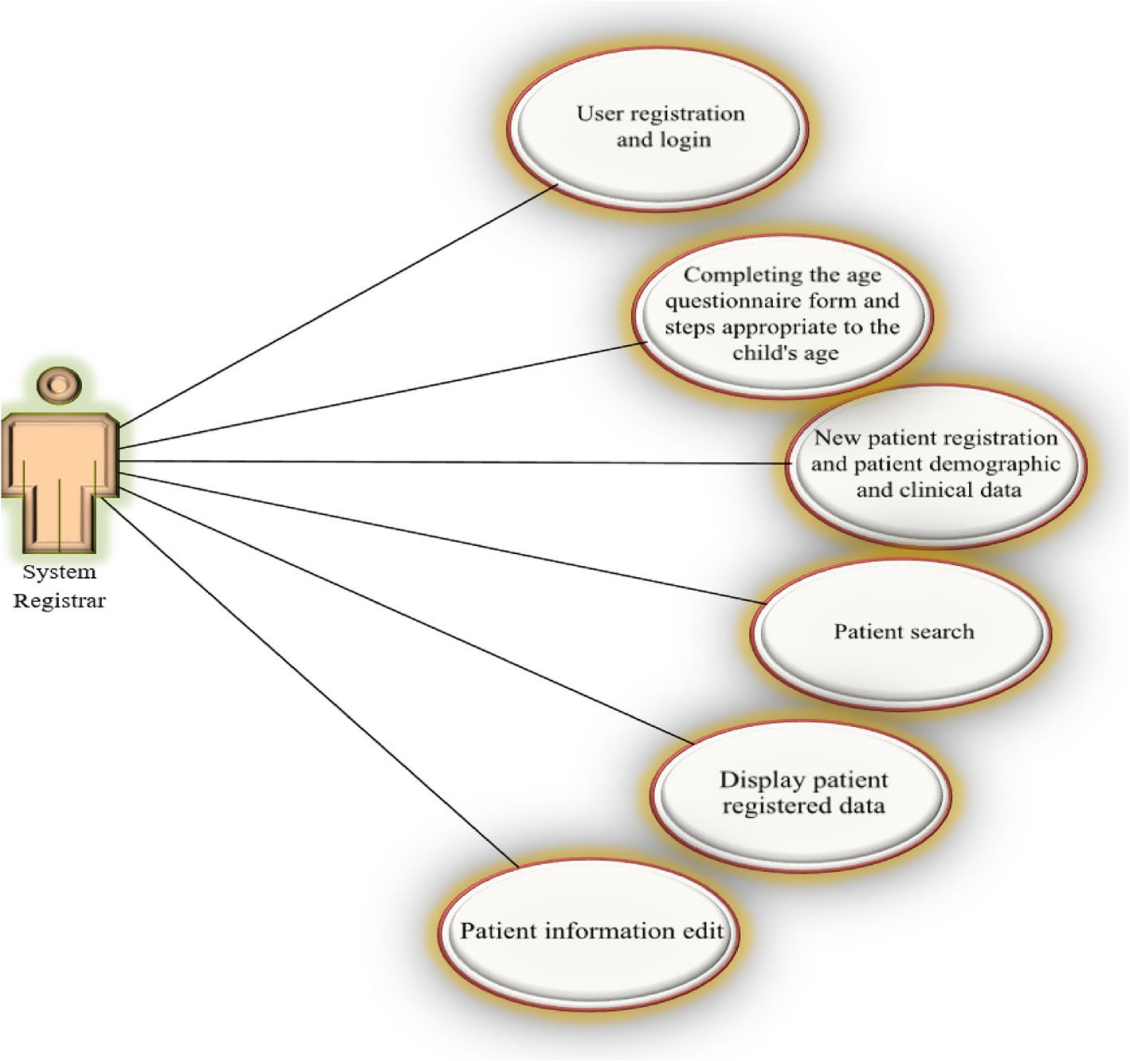
User diagram of the system registrar

**Fig. 4:**
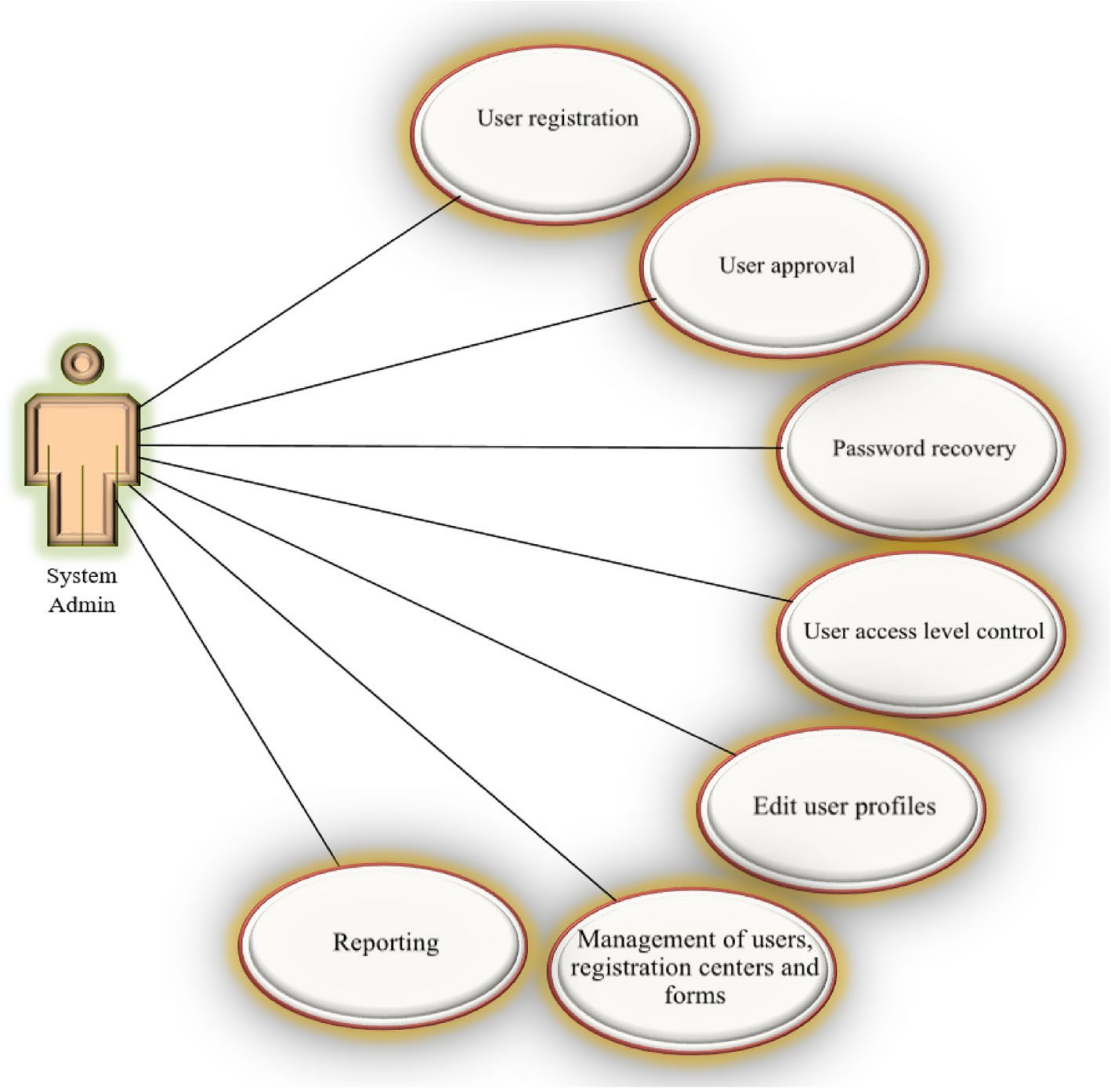
User diagram of the system admin

**Fig. 5:**
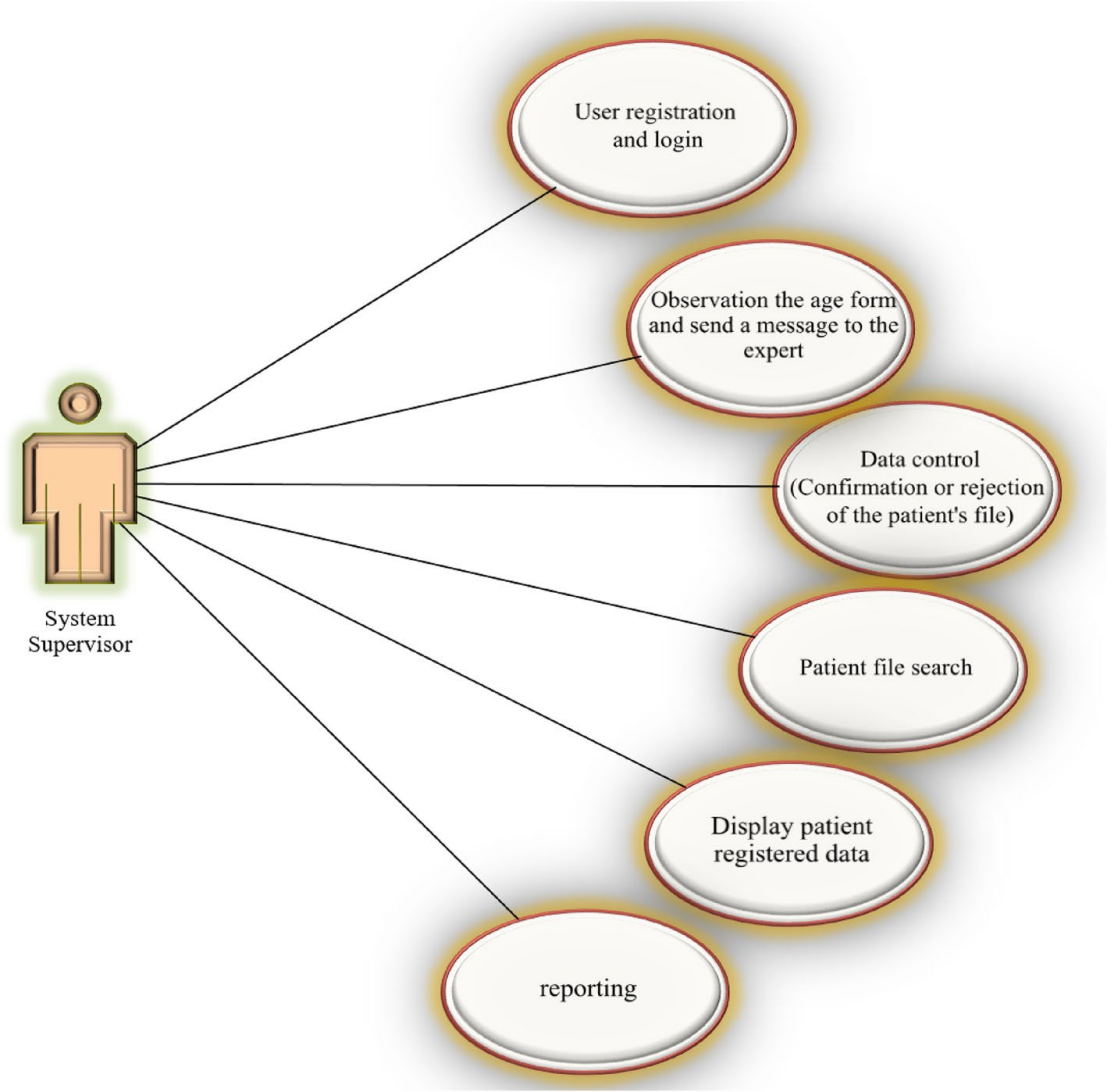
User diagram of the system supervisor

The system of registering and monitoring children's developmental motor disorders is connected with three actors, the user who registers information, the system administrator, and the system supervisor, and provides them with facilities according to the defined roles and access levels. The diagrams used by the three actors are shown in Figs. 3, 4 and 5.

The clients and the system server are connected through a university physical network's IP/TCP communication protocol. The registering and monitoring system of children's developmental motor disorders is web-based, so the ASP.NET framework was used on the server. This system was designed in Visual Studio-2018 software, and C# programming language was used in the NET 4.5 technology platform for high-level programming. SQL Server database was used to store data. Since this system is web-based and does not require software installation, users can access the system through all browsers after specifying the username and confirming access.

**Fig. 6:**
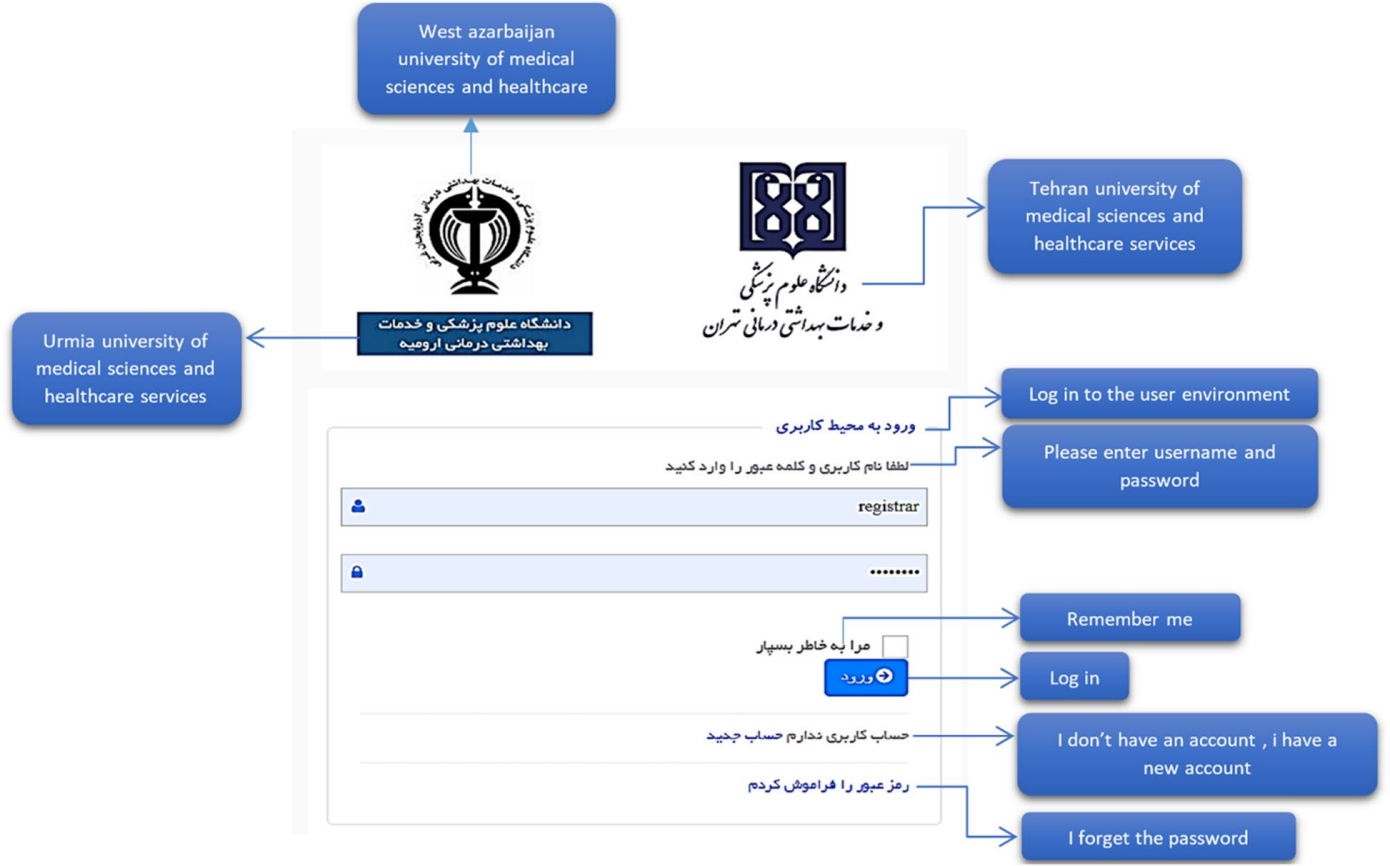
Login page to the system registrar

**Fig. 7:**
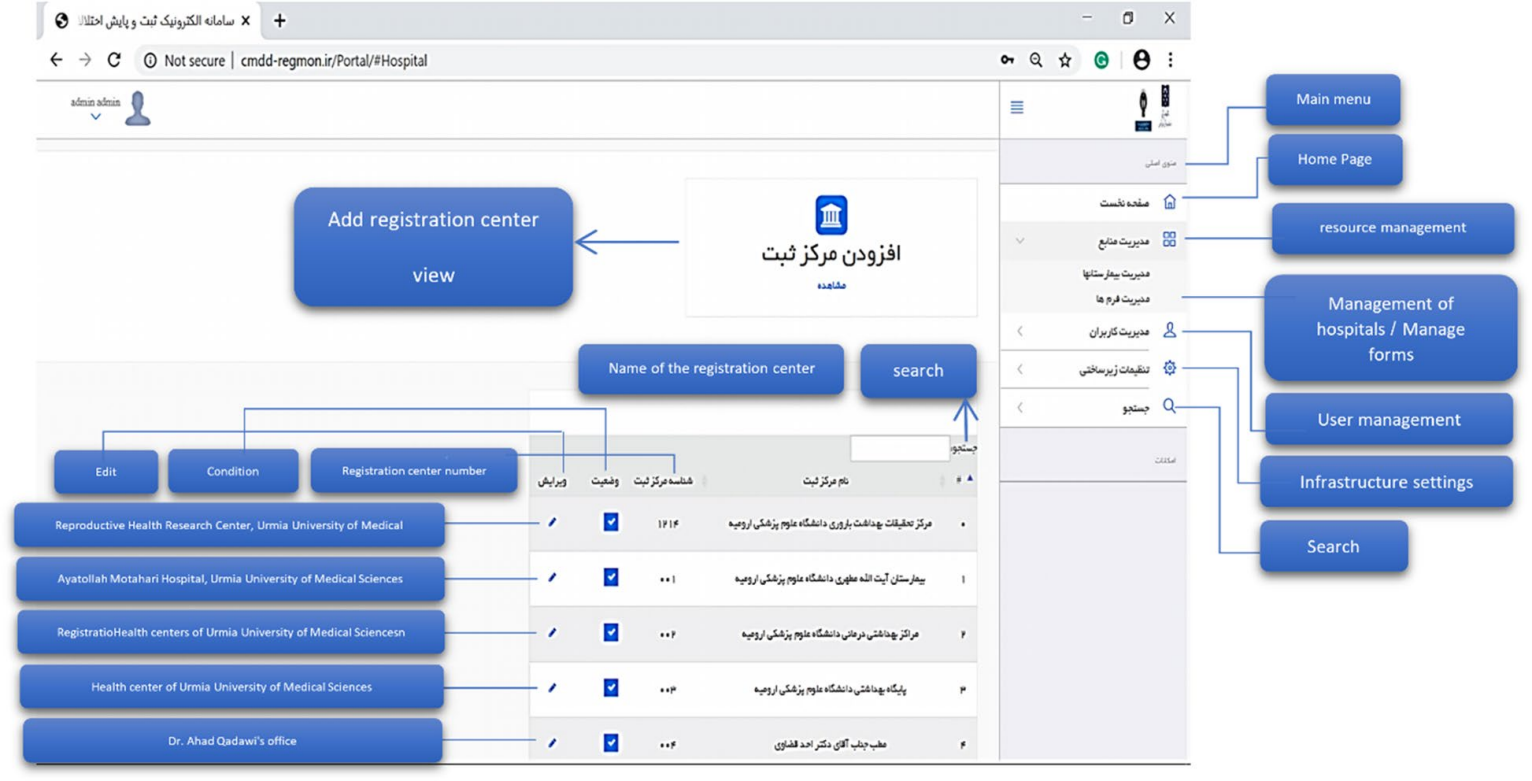
Management of registration centers in the system administrator environment

**Fig. 8:**
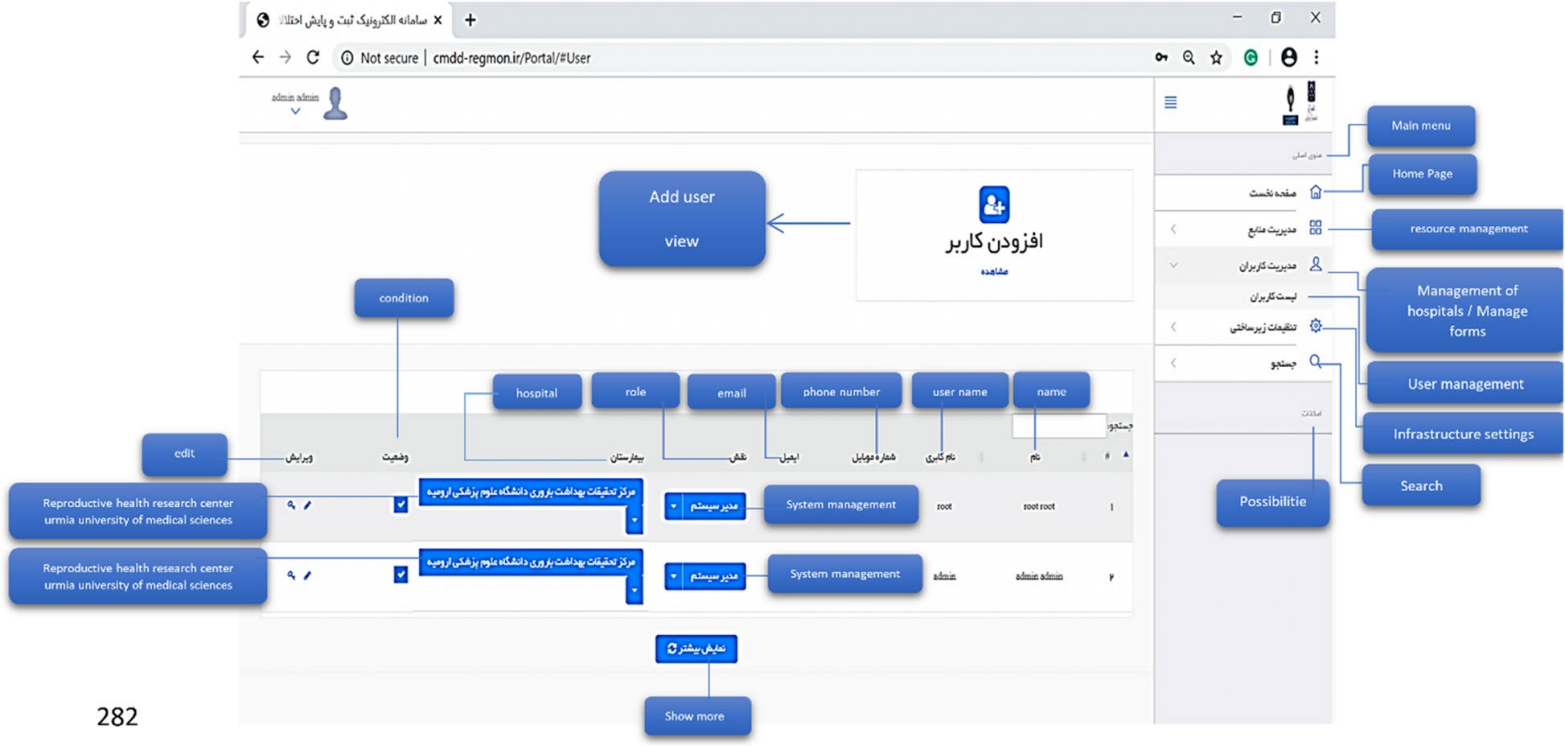
User management in the system administrator environment

**Fig. 9:**
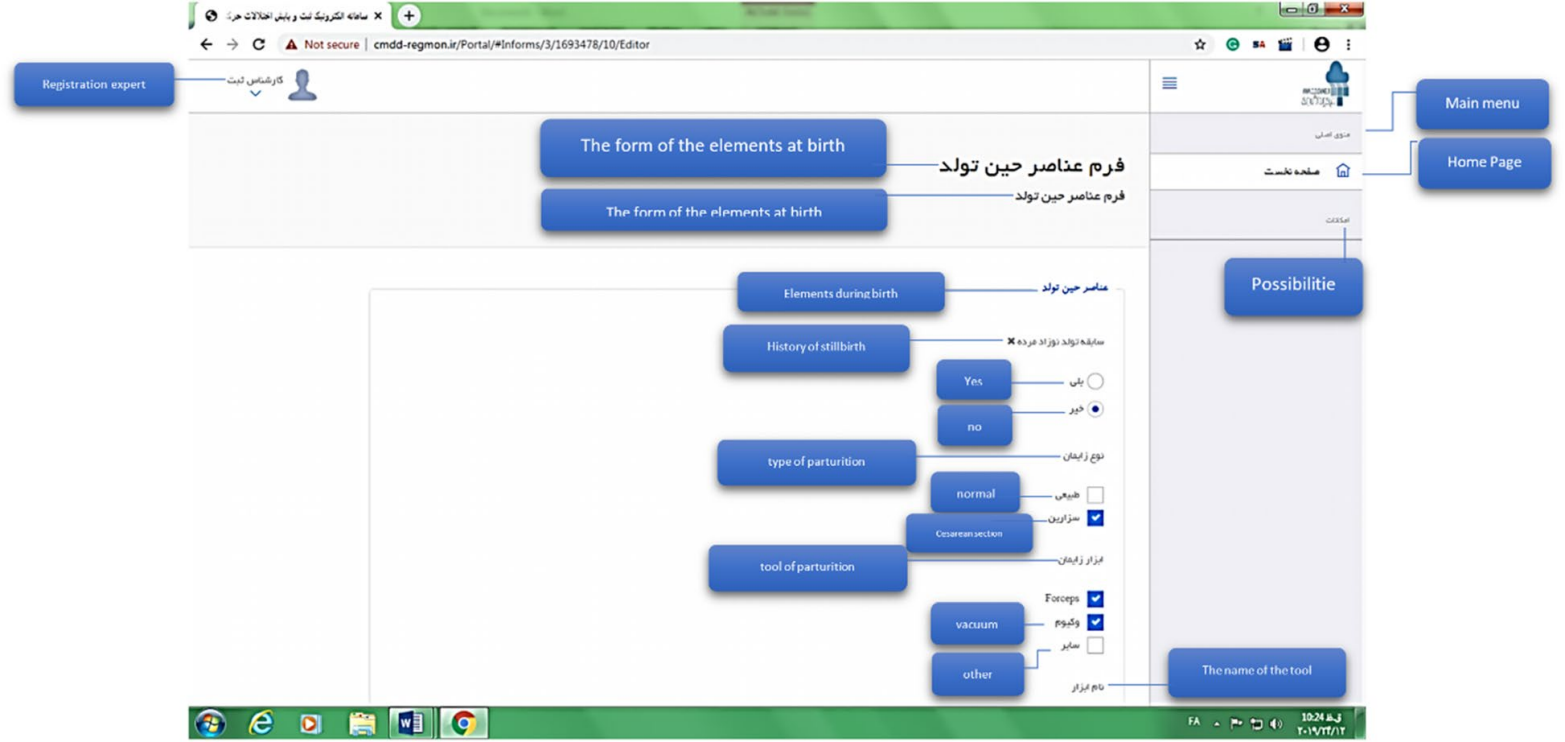
A filled form by registrars

**Fig. 10:**
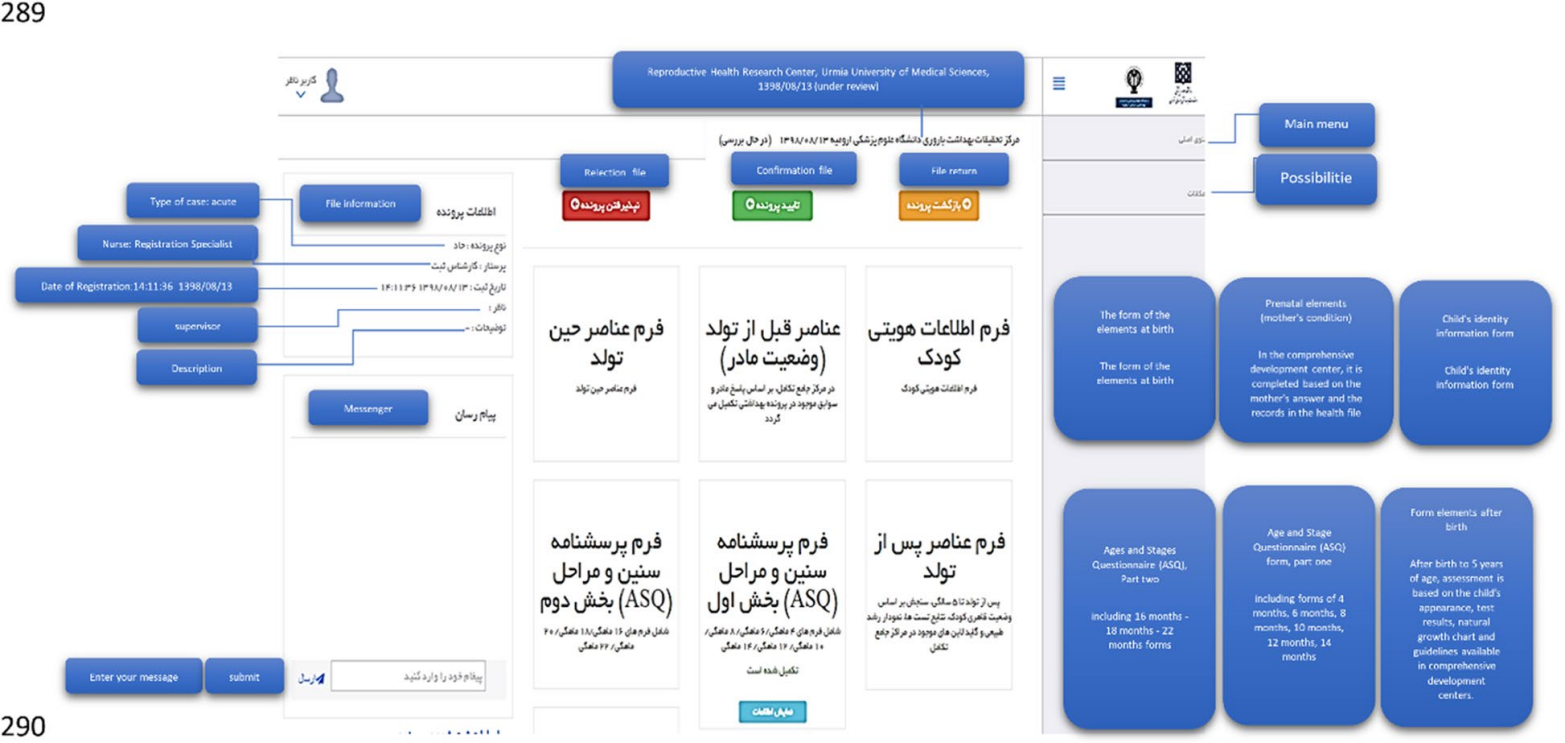
User environment of Supervisor

The electronic system of registering and monitoring children's developmental motor disorders has three actors: Admin, Registrar, and Supervisor. Admin administrates the system. Admin checks and approves the requests to use the system and defines users' right of access to different parts of the system based on role. A registrar is a regular user who enters information related to forms. A supervisor is a user who controls and approves the information registered by the registrar. An example of the user login page for the registrar is shown in Fig. 6. As mentioned, three actors are defined for the system, and each of them has its user environment. For example, Fig. 7 shows an example of the system admin work environment related to the registry center management section. In this section, the admin defines the centers with permission to register information for the system and gives access to their users (Fig. 8). Registrators use the system to register patient and mother information in various forms. They can also receive important reminders to adhere to the treatment. An example of registrator information registry forms is shown in Fig. 9. As mentioned, the supervisor also has its own unique user interface and access level. For example, after entering the user environment, he can search and control the registered records (Fig. 10).

After designing the system, it was implemented as a pilot in the specialized clinic for children's growth and development. The study's statistical population in the evaluation phase was 14, and the response rate was 100%. The frequency of the evaluation results is presented in Tables 1, 2, 3, 4, 5 and 6. The mean results of data content evaluation, physical system design, flexibility, technical characteristics, system participation, and user support were obtained at 88.35%, 84.87%, 89.96%, 89.7%, 85.68%, and 84.3%, respectively.

**Table 1:** The results of evaluating the data content of the system

Group	Main criteria	Frequency (Percentage)	
		NO	Yes
Data content	The data content is consistent with the system's objectives	3 (21/5)	11 (78/5)
	The content and context of the data elements are compatible with the system theme	1 (7/2)	13 (92/8)
	The system's data elements are clear, appropriate, and not ambiguous	2 (14/3)	12 (85/7)
	The patient data elements included in the system are complete and appropriate	1 (7/2)	13 (92/8)
	The disease data elements included in the system are complete and appropriate	3 (21/5)	11 (78/5)
	The data elements related to the history included in the system are complete and appropriate	1 (7/2)	13 (92/8)
	Data elements related to clinical evaluations included in the system are complete and appropriate	0	14 (100)
	The data elements related to therapeutic interventions included in the system are complete and appropriate	2 (14/3)	12 (85/7)

**Table 2:** The results of evaluating the physical design of the system

Group	Main criteria	Frequency (Percentage)	
		NO	Yes
Physical design	The system design is clear and understandable for users	1 (7/2)	13 (92/8)
	The design of all system pages is compatible with each other	3 (21/5)	11 (78/5)
	The system's design seems logical from the users' point of view	4 (28/6)	10 (71/4)
	The design of the system is such that it guides users to find what they want	1 (7/2)	13 (92/8)
	The system can search on the patients' page	0	14 (100)
	The graphics of the system are well designed	3 (21/5)	11 (78/5)
	The background and texts of the system are well designed and easy to read and understand	2 (14/3)	12 (85/7)
	The colors used in the system are suitable and effective	3 (21/5)	11 (78/5)
	The font size of the content in the system is suitable for different users	2 (14/3)	12 (85/7)

**Table 3:** System flexibility evaluation results

Group	Main criteria	Frequency (Percentage)	
		NO	Yes
Flexibility	Different parts of the system have clear and correct titles for different users	0	14 (100)
	The content of the system has suitable adaptability	2 (14/3)	12 (85/7)
	While the system has a unique style, it serves different levels of users	1 (7/2)	13 (92/8)
	Different parts of the system have the flexibility to interact with users	3 (21/5)	11 (78/5)
	In order to increase flexibility, it is possible to access a specific page from different paths	1 (7/2)	13 (92/8)

**Table 4:** Evaluation results of technical features of the system

Group	Main criteria	Frequency (Percentage)	
		NO	Yes
Technical features	All the links in the system pages work properly	0	14 (100)
	System pages are supported in conventional browsers, including Internet Explorer	1 (7/2)	13 (92/8)
	All multimedia resources of the system are available and working at any time	2 (14/3)	12 (85/7)
	Users can easily access the system's website at any time	1 (7/2)	13 (92/8)
	There are complete and clear paths for installing or accessing the system	1 (7/2)	13 (92/8)
	It is possible to receive output in conventional formats, including Excel in the system	3 (21/5)	11 (78/5)
	It is possible to search for records based on the patient's first and last name and national code	2 (14/3)	12 (85/7)

**Table 5:** The results of the evaluation of system participation

Group	Main criteria	Frequency (Percentage)	
		NO	Yes
Participation	The system forces users to participate in the processes actively	2 (14/3)	12 (85/7)
	The visual features of the system are suitable for increasing the motivation of users to continue working with the system	4 (28/6)	10 (71/4)
	Users can receive appropriate feedback while working with the system	1 (7/2)	13 (92/8)
	Working with the system is not boring for users	0	14 (100)
	Working with the system is simple and does not require much training	3 (21/5)	11 (78/5)

**Table 6:** Evaluation results of system user support feature

Group	Main criteria	Frequency (Percentage)	
		NO	YES
User support	The system provides ways for technical support to users	4 (28/6)	10 (71/4)
	The system provides external resources, including some appropriate websites, to the user	0	14 (100)
	The system's content and data leveling are appropriate to its users' ability	1 (7/2)	13 (92/8)
	In order to perform tasks by users, the defined paths are clear and complete enough	2 (14/3)	12 (85/7)
	Features such as notice boards and sending comments for users are embedded in the system	4 (28/6)	10 (71/4)

The general results of the evaluation of the electronic system of registering and monitoring children's developmental motor disorders, both in terms of the adequacy and appropriateness of the information content and in terms of the software quality, showed that the current system had been approved by its users with an agreement rate of 87.14% and it could create an acceptable level of satisfaction in this stage of the work.

## Discussion

Collecting real-life data of patients is a critical step in patient management and clinical science. In the past, patients' information was registered manually. The number of qualitative registries is increasing with the implementation of electronic patient registry systems that allow for easier and faster data registration. Well-designed registry systems are a good way to collect and analyze disease data in a real environment and have added value in randomized controlled studies [26]. They can also represent clinical practice, disease outcomes, safety, and effectiveness [27]. The first step in designing a registry system is to determine the requirements. As mentioned, the results of this step of the present study have been published in the previous article [22].

After determining the requirements for designing a system, conceptual modeling is done. Conceptual modeling is used to identify, analyze, describe, and graphically display the concepts and limitations of a field [28]. Since analyzing healthcare systems is complex and challenging, UML is used to analyze healthcare systems [29]. Unified modeling language specifies, visualizes, constructs, and documents software systems and facilitates communication between project members. The unified modeling language is more than a few graphical symbols; manufacturers can create models that other manufacturers can understand with the help of it [28].

Naemi et al. used UML diagrams before creating the Web-Based Dental Implant Registry (DIR) system to consider the appropriate system according to the teeth' anatomy to meet dentists' needs [30]. In the present study, scenarios, tables, and diagrams of the unified modeling language were used to define the structure, behavior, and interaction of users with the system to create suitable conditions for modeling the information flow and facilitating the correct creation of the system. This approach is a suitable tool for modeling healthcare information processes and the future development of systems. It creates a common language among the analysts, developers, and users of systems. Thus, the needs and demands of the users, as well as the requirements of the system, should be clearly displayed in the design of health information systems using the capabilities of the unified modeling language diagrams so that the system developers can identify their user needs and requirements to design and implement information systems.

After conceptual modeling, the output of the next step was a web-based system that registers the information of children with developmental motor disorders to manage the condition of these patients effectively. Web-based systems are more popular to their user-friendly design and access without time and place restrictions. These systems increase the accuracy and speed of data registration and reduce possible errors. Moreover, their use facilitates updating information, creating complex reports, and sharing information [31, 32]. Studies in different countries suggest that most of these systems are web-based. For example, in a study entitled "Development of the cerebral palsy follow-up registry system," Almasri et al. [33] in Jordan designed a web-based registry system intending to register the follow-up of children with cerebral palsy.

This system provides demographic information about the child and parents and distributes the participants based on topography. In other clinical areas, web-based registry systems have been designed. For example, the Web-Based Dental Implant Registry (DIR) system was developed in Naemi et al.'s study [30].

In the present study, all demographic data elements and clinical-prenatal data related to mother and child are collected [22]. In addition to registering the information of children with cerebral palsy, this study has the potential of registering the information of children suffering from all developmental motor disorders. After creating the system of registering and monitoring children's developmental motor disorders, this system was evaluated in two steps. In the first step, the accuracy and correctness of the system performance was evaluated by the researcher, and the system faults and errors were fixed and the system correctness was confirmed. In the next step, the system was assessed from the end users' viewpoint. By evaluating health information systems, it is possible to ensure that inappropriate results are corrected. There are various methods for assessing information systems [34]. Evaluation from the usability viewpoint is one of these methods in which the system helps users perform their tasks to do their work quickly and with minimum effort [35]. Usability is directly related to errors, productivity, and user's satisfaction [36], so low system usability reduces information usage by users [35].

In information systems, many errors are related to usability problems; for example, incorrect and ambiguous messages, unfamiliar and complex language, and feedback lacking important information reduce efficiency and confuse users. Usability problems of health information systems can affect the quality of users' interaction with the system and, thus, the care outcome. The evaluation of the usability of the information system will be effective in identifying and solving these problems [37]. The present study evaluated the system by focusing on usability and user satisfaction. It obtained an acceptable score in all areas, including information content, physical design, flexibility, technical capabilities, participation, and the level of user support. The general results of the system evaluation in terms of the adequacy and appropriateness of the information content and also in terms of software quality show that the system is approved by its users with an agreement rate of 87.14% and could create an acceptable level of satisfaction in this stage of the work.

Sadeqi Jabali et al. evaluated the medical records and admission information system using the usability evaluation method to identify system problems. In this study, 62 unique usability problems were identified. The highest number of problems was related to the two principles of "compliance with uniformity and standards" and "diagnosis instead of reminding," and the highest mean severity of problems was related to the two principles of "clarity of system status" and "aesthetic aspects and convenient design" and the lowest mean severity of problems was the principle of " error prevention" [38]. In the study by Naemi et al., the dental registry system was evaluated in terms of usability. In the mentioned study, the largest number of problems was related to system clarity and adaptability principles between the system and the real world [30]. The concept of usability in health is more crucial than in other areas because it deals with the quality of care, patient safety, and human and social issues. Thud, it is recommended to evaluate its user interfaces in the following stages, such as the development and implementation stages of the system. These continuous evaluations significantly reduce system usability problems and costs, improving user interaction and care outcomes.

## Conclusion

The output of the present study was an electronic system of registering and monitoring children's developmental motor disorders. It was an attempt to combine the results of the review of relevant documents and articles in other countries with the suggestions and opinions of experts and present an acceptable product based on the available facilities in the country. By providing the necessary conditions for the ease of access of service providers to the data of patients with motor disorders to identify affected patients and groups at risk, this study's results can be valuable and effective in treatment, reducing consequences, economic costs, optimal allocation of resources and in planning and the decisions of the country's policymakers. It also has many benefits, including registering and monitoring to improve the efficiency of disease surveillance for use in health planning, management, and evaluation of control strategies for taking effective and timely measures. Also, implementing such a system can be valuable for all kinds of studies and a helpful tool for research.

Due to a lack of information integrity, inconsistency, and lack of communication between the relevant organizations in charge of providing services in Iran, such children and their mothers' information is not manually registered completely and timely. Although the creation of this system solves the problems to some extent, it requires the participation of all healthcare providers, including specialists, physicians, and other health service providers. Also, there is no registering and monitoring system for children's developmental motor disorders in Iran, and all the systems created in the form of different diseases operate individually. Hence, creating and applying a national children's developmental motor disorder registry system is a wise investment to improve the understanding of these diseases, provide better services, and facilitate related research. Also, if registry systems are properly created and integrated with other care systems such as electronic health records, it will have many advantages such as better management of resources, improvement of care coordination, effective management of diseases, national and international access to clinical data, reduction of medical errors, prevention of delay in treatment of patients, reduction of operating costs and increase of patients' participation in the treatment.

The results of the present study can be used as a road map for implementing the national system of developmental movement disorders of children in the country. In the future, this system can be connected with other related information systems to create a comprehensive database and facilitate information sharing of children with developmental movement disorders. It is also possible to plan in the future to create self-care applications based on mobile phones for patients with developmental movement disorders children that are also connected with this system.

One of the limitations of this research was access to the full text of some relevant articles, which was tried to be solved by emailing the authors. Also, the number of available specialists who worked in the clinic to evaluate the system was low, which was tried to be compensated by diversity in expertise from other cities.

## Supplementary Information

**Additional file 1.** 

## Data Availability

The datasets used and/or analyzed during the current study are available from the corresponding author on reasonable request.
