# Supplementary material for: Designing and evaluating the children’s developmental motor disorders system: an experience from a developing country
Source: BMC Med Inform Decis Mak. 2023 Jul 17;23:123. doi: 10.1186/s12911-023-02223-2 (PMC10351156; doi:10.1186/s12911-023-02223-2)
Supplement: Supplementary file 1 — Additional file 1. [file 12911_2023_2223_MOESM1_ESM.docx]

**Questionnaire**

**Evaluating the data content of the system**

| **Response** | | **Main criteria** | **Group** |
| --- | --- | --- | --- |
| **No** | **Yes** |  |  |
|  |  | The data content is consistent with the system's objectives. | **Data content** |
|  |  | The content and context of the data elements are compatible with the system theme |  |
|  |  | The system's data elements are clear, appropriate, and not ambiguous. |  |
|  |  | The patient data elements included in the system are complete and appropriate. |  |
|  |  | The disease data elements included in the system are complete and appropriate. |  |
|  |  | The data elements related to the history included in the system are complete and appropriate. |  |
|  |  | Data elements related to clinical evaluations included in the system are complete and appropriate. |  |
|  |  | The data elements related to therapeutic interventions included in the system are complete and appropriate. |  |
|  |  | The system design is clear and understandable for users. | **Physical design** |
|  |  | The design of all system pages is compatible with each other. |  |
|  |  | The system's design seems logical from the users' point of view. |  |
|  |  | The design of the system is such that it guides users to find what they want. |  |
|  |  | The system can search on the patients' page. |  |
|  |  | The graphics of the system are well designed. |  |
|  |  | The background and texts of the system are well designed and easy to read and understand. |  |
|  |  | The colors used in the system are suitable and effective. |  |
|  |  | The font size of the content in the system is suitable for different users. |  |
|  |  | Different parts of the system have clear and correct titles for different users. | **Flexibility** |
|  |  | The content of the system has suitable adaptability. |  |
|  |  | While the system has a unique style, it serves different levels of users. |  |
|  |  | Different parts of the system have the flexibility to interact with users. |  |
|  |  | In order to increase flexibility, it is possible to access a specific page from different paths. |  |
|  |  | All the links in the system pages work properly | **Technical features** |
|  |  | System pages are supported in conventional browsers, including Internet Explorer |  |
|  |  | All multimedia resources of the system are available and working at any time |  |
|  |  | Users can easily access the system's website at any time |  |
|  |  | There are complete and clear paths for installing or accessing the system |  |
|  |  | It is possible to receive output in conventional formats, including Excel in the system |  |
|  |  | It is possible to search for records based on the patient's first and last name and national code |  |
|  |  | The system forces users to participate in the processes actively. | **participation** |
|  |  | The visual features of the system are suitable for increasing the motivation of users to continue working with the system. |  |
|  |  | Users can receive appropriate feedback while working with the system. |  |
|  |  | Working with the system is not boring for users. |  |
|  |  | Working with the system is simple and does not require much training. |  |
|  |  | The system provides ways for technical support to users. | **User support** |
|  |  | The system provides external resources, including some appropriate websites, to the user. |  |
|  |  | The system's content and data leveling are appropriate to its users' ability. |  |
|  |  | In order to perform tasks by users, the defined paths are clear and complete enough. |  |
|  |  | Features such as notice boards and sending comments for users are embedded in the system. |  |
